# Determinants of safe sexual behavior of female sex workers in Tehran: the woman, her network, and the sexual partner

**DOI:** 10.1186/s12889-021-12266-7

**Published:** 2021-12-06

**Authors:** Zahra Jorjoran Shushtari, Yahya Salimi, Seyed Ali Hosseini, Homeira Sajjadi, Tom A. B. Snijders

**Affiliations:** 1 Social Determinants of Health Research Center, University of Social Welfare and Rehabilitation Sciences, P.O Box: 1985713834, Tehran, Iran; 2 Social Development and Health Promotion Research Center, Health Institute, Kermanshah University of Medical Sciences, Kermanshah, Iran; 3 Department of Sociology, University of Groningen, 9712 TG Groningen, Netherlands; 4 Nuffield College, University of Oxford, Oxford, OX1 1NF UK

**Keywords:** Safe sexual behavior, HIV/AIDS, Female sex workers, Social network

## Abstract

**Background:**

Despite the steady growth of sexual transmission of HIV, there is little evidence about safe sexual behavior of FSWs, and social network effects on this behavior, in Iran. Our aim in this study was to determine the effect of social network characteristics on condom use among FSWs, considering individual characteristics of the FSWs and of their sexual partners, characteristics of their relationship, and the FSW’s personal network.

**Methods:**

A cross-sectional ego-centric network survey of 170 FSWs was carried out in Tehran between January and June 2017. A multilevel ordered logistic regression analysis was conducted to examine the effects of individual and relational characteristics simultaneously.

**Results:**

Condom use in sexual relationships of the FSWs on average was rather low. Important determinants of safe sexual behavior were found both at the level of the individual FSW and at the level of the sexual partner. The main determinants at the level of the individual FSW were FSWs’ age and HIV knowledge. At the level of the sexual partner, age and education of sexual partners, as well as intimacy, duration of tie, frequency of contacts with a given partner, frequency of contact, perceived social support, and perceived safe sex norms were significantly associated with condom use.

**Conclusions:**

The findings highlighted that considering only the individual characteristics of female sex workers is not sufficient for effectively promoting condom use. Factors at the network and dyadic level should also be considered, especially the role of sexual partners. Network-based interventions may be useful which modify social relationships to create a social environment that can facilitate changes in sexual behavior.

## Introduction

Prevention of Human Immunodeficiency Virus (HIV) infection is currently one of the most important global health concerns. Globally, in 2018, 37.9 million people had HIV, of which 1.7 million were new HIV infections [1]. Despite recent progress in HIV prevention [2, 3], HIV prevalence has an increasing trend in Iran [4, 5].

According to the last UNAIDS report, 61,000 people were living with HIV in 2018 in Iran [6]. In recent years, patterns of HIV transmission in Iran have been changing. Although injection drug use remains the main cause of HIV transmission, sexual transmission has been steadily growing (from 7% in 2006 to 33% in 2012, and to 42% in 2016). Prevalence of HIV among female sex workers (FSWs) was estimated to be 4.5% in 2014 [4, 7, 8]. A national behavioral survey conducted in 2010 throughout 14 cities in Iran reported that HIV prevalence among FSWs was 4.5% (95% CI=2.4 to 8.3), 4.8% (95% CI=2.2 to 9.8) among those who had reported a history of drug use and 11.2% (95% CI=5.4 to 21.5) among those who had a history of injection drug use [9].

Female sex workers are a hidden and under-reported population whose risk behaviors such as not using condoms, having multiple partners, using drugs or alcohol before sexual relationships, and intravenous drug use, can contribute to an increased rate of HIV transmission [10]. They engage in sexual relations in exchange for something else such as money, drugs, gifts, and shelter. They are not only themselves at risk for HIV, but also may be a bridging group that may transmit HIV to the general population. According to a size estimation study, there were 228,700 FSWs (95% CI: 153,500–294,300) in Iran in 2012 [7]. However, there is little information about this vulnerable subpopulation and their HIV risk behaviors, due to the fact that prostitution is considered as taboo by both government and the general population, and goes along with high social stigma. According to previous studies in Iran, most FSWs knew that condom use is a method to reduce risk of HIV transmission, but only few of them used condoms with their paying and non-paying partners [11].

Although there are some studies about FSWs in Iran as well as other countries, most of them have focused on assessing individual characteristics of FSWs such as HIV knowledge, attitude, risky sexual behaviors, and sexually transmitted disease status [11–13]. However, the FSW herself represents only half of a sexual dyad. Considering the characteristics of only one member of the dyad does not provide sufficient information about the social context of HIV-related risk behaviors and the quality of interpersonal interactions that may facilitate those behaviors and health outcomes.

There is ample evidence that social networks have an important role generally for risk and health behaviors by providing opportunities for social influence, social support, and social engagement [14, 15], and also specifically for sexual behaviors [16]. There are several Several studies which were conducted among vulnerable population such as injecting drug users (IDUs) [17], men who have sex with men (MSM) [18], and adolescents or youth who use drug or alcohol [19], showed that perceived norms are associated with risk behaviors. Also, previous studies showed that social support from network members, especially gatekeepers (managers or pimps) and peers, was significantly associated with condom use among FSWs [20, 21].

Despite the advances in our understanding about the potential role of social networks for HIV risk behaviors, our systematic review study showed that evidence about effects of social networks on HIV risk behavior of FSWs is scarce [22]. Most existent studies only considered a few network-related characteristics such as frequency of contact, trust, and social support among FSWs [21, 23–28]. Therefore, a comprehensive study to clarify the mechanisms according to which social network characteristics of FSWs affect their HIV risk behaviors is greatly warranted.

Our study is about sexual behavior of FSWs, which takes place in the sexual network made up by the FSW and her sexual partners including her clients and regular partners. When studying network effects on this behavior, factors at the level of the individual may be distinguished from those at the network level. The aim of this study was to determine the effect of social network characteristics on the extent of condom use by FSWs in Tehran, considering individual characteristics of the FSWs and of their sexual partners, characteristics of their relationship, and of the FSW’s personal network.

## Methods

### Setting

This study is a cross-sectional ego-centric network survey of 170 FSWs in Tehran city, conducted between January and June 2017. An ego-centric network survey provides more insight into the effects of the social network around the FSW on her condom use by making an inventory of the personal network of the FSW and collecting relevant individual characteristics of the FSW herself as well as of her sexual partners. This purpose is achieved using a so-called name generator for making the inventory of the members of the personal network, and a name interpreter for determining their relevant characteristics and relevant interactions. In our study, the FSW who is the respondent is the focal individual (ego) in the personal network.

### Participants

FSWs are a hard to reach population in Iran, subject to social and official taboos and social stigma. However, in recent years enormous governmental and non-governmental investments and efforts have been made to increase the access of this vulnerable population to HIV prevention programs, such as the creation of drop-in centers (DICs) and consultation centers for at risk women throughout the country. These centers provide free services including basic sexual and reproductive health care, educational programs about sexual transmitted infections (STI) and prevention methods, HIV testing, and counselling [29, 30].

It is virtually impossible to obtain a random sample from a hidden and stigmatized population such as this. Therefore, participants were recruited through snowball, purposeful, and convenience sampling methods as successful alternatives to non-applicable random sampling methods for recruiting ‘hidden’ populations [31–33]. Eligibility criteria were being over 16 years old, having had sex for money, drugs, and so on in the last year, identifying oneself as a sex worker, and willingness to participate in the study. For the snowball sample, to initiate the chain referral process, six FSWs who satisfied the inclusion criteria were selected as index participants. After interviewing the index participants, we asked them to introduce some other sex workers whom they had named as their network members. The snowball sample proceeded according to the principles of respondent-driven sampling [31]. We gave the respondents some coupons for their peer friends, which included the ID number, aim of the study, amount of incentive, place of visit for interview, and an expiration date. Each participant was given 100,000 Rails (equal to 3 USD) as a small primary incentive for participating in the study and completing the interview; and a secondary small incentive, of the same amount, if they had introduced their peer friends, who did sex work and named as their network members. This process continued until five waves. In all waves the participants introduced few peer friends. Some introduced only one or two peer friends named as network members, some others introduced other FSWs who had not been named as their network members. It was impossible to access all peer members who were named by the participants. We completed the sample with convenience sampling and purposeful sampling methods. Convenience sampling was used by recruiting participants among FSWs who attended DICs and consultation centers. Recruitment of study participants in DICs was facilitated by DIC staff, who were personally contacted at the center by the first author. The DIC staff identified potential participants and introduced them to the interviewer. To improve diversity from all involved sites, we also used purposeful sampling from outreach spots such as team homes, streets, and parks by a peer outreach worker who collaborated with the study via a DIC. To maintain anonymity of the FSWs, we received verbal informed consent from all participants, because sex work is illegal in Iran. We provided an explanation regarding the study purpose to all participants, and informed them about the confidentiality and their right to withdraw from the study at any time during the interview.

### Measurements and variables

The data were gathered using an investigator-constructed questionnaire, after assessing content validity, scalability, and reliability. The questionnaire consisted of two parts, individual and network information. Individual information was collected about the demographic characteristics age, educational level, marital status, and place of living, and about the frequency of sex work in the last month, HIV knowledge, and HIV test. For network information, first, a name generator inventory [34] was applied to indicate the FSW’s sexual network members. The sexual network was defined as the set of nominated persons with whom they had had sex in the past 30 days. We asked the FSWs to nominate up to 5 persons with whom they had any sexual relationships during the past 30 days.

For these nominated persons further information was collected including socio-demographic information, duration of contact, frequency of contact, frequency of condom use, intimacy, social support, and drug or alcohol use before a sexual relationship with him. The data was collected in face-to-face structured interviews by trained peer interviewers, which was helpful to build trust and get honest responses from the participants. Each interview usually lasted around 45 minutes.

### Socio-demographic variables

The socio-demographic variables were collected as follows. Age was recorded in years. Educational level was measured in six ordinal categories, coded as 1 to 6: illiterate, able to read and write, primary education, secondary education, high school or diploma, and university education. Marital status was measured in four categories as single, married, divorced, and widowed; for the analysis this was dichotomized into never married and ever married. Place of living was categorized as homeless, living in the home of others, and personal home. The number of people supported by the FSWs was measured as a count; for the analysis it was dichotomized as zero versus more than zero. The variables collected for the network members were age and educational level, measured similarly as for the respondents.

### Social network variables

To assess the frequency of contact with the sexual network members, we asked the participants “How many times did you meet him or did you communicate with him in the last month?”. To assess duration of the tie we asked the participants “Since how long have you known him?”. This was coded in months. The intimacy of the relationship between participants and sexual network members was assessed with a five-point Likert response, with categories very close, close, somewhat close, distant, and very distant. Social support was assessed by an investigator-constructed questionnaire with five items. Reliability of the items comprising the social support questionnaire was pilot-tested prior to final implementation in this study. Cronbach’s alpha and intraclass correlation for the scale were .82 and .85, respectively. Mokken scale analysis was used to assess scalability and uni-dimensionality of the social support questionnaire [35]. The Loevinger *H*-coefficient for the questionnaire was larger than 0.5, characterizing it as strong scale.

Because sexual network members could be nominated for more than one role or interaction (a sexual partner may also have a familial tie and/or be a drug use partner), the multiplexity of their position was determined. This was defined as 1 if they had more than one role in the respondent’s network, and 0 if they had just one role in her network (i.e., only sexual partner). The density of each network was also assessed. To assess density, participants were given a matrix with the names of their mentioned sexual network members on both axes, and then were asked to indicate the pairs of network members who knew each other. The density was calculated as the number of sexual network members who knew each other divided by the maximum number possible, given the size of the sexual network.

The variable of drug and alcohol use before or with sex was dichotomized into “yes=1”, and “no=0”.

### Perceived safe sex norm

Perceived safe sex norms were assessed by the perceived norm scale (PNS) of the safe sex norm questionnaire (SSNQ) [16]. The PNS assesses perceptions of the FSW regarding attitudes and behaviors about condom use of their peer friends who do similar sex work. It uses 17 questions each on a five-point Likert scale: “all”, “most”, “about half”, “some” and “none”. These were coded as scores from 1= “none” to 5= “all”. The reliability of the questionnaire was pilot-tested. The attainable score range is 17-85. Higher scores indicate that the perception of FSWs of the attitude and behaviors of their friends is that most of them have a positive attitude about condom use and frequently use it in their sexual relationships [16]. Cronbach’s alpha and the intraclass correlation for this scale were 0.89 and 0.83, respectively. Mokken scale analysis was used to assess scalability and uni-dimensionality of the questionnaire [35]. The Loevinger *H*-coefficient for the scale was larger than 0.5, characterizing it as strong scale.

### HIV Knowledge

Knowledge about HIV transmission was evaluated by a 14-item tool based on an established questionnaire in the Iranian population [36]. The questionnaire was pilot-tested with 28 participants in Tehran. Scalability and uni-dimensionality of the questionnaire were assessed by Mokken scale analysis [35]. Each item and the whole questionnaire had Loevinger H coefficients above 0.4, which is good. Cronbach’s alpha for this scale was 0.86. The sum score was transformed to a scale of 0 to 100, with high scores meaning more knowledge.

### Sexual practice and HIV test

For assessing sexual practices, the participants were asked to report about the frequency of sex work in the last month. This was recorded as a count variable. Participants were also asked to report if they ever had a HIV test. This, together with the test result, was recorded as a categorical variable with values “having HIV test & positive result=1”, “having HIV test & negative result or don’t know=2”, and “having no HIV test=3”.

### Condom use

Frequency of condom use by participants and the sexual network member was the dependent variable, measured on a five-point ordinal scale. It was defined as *Yij* = 1 if for network member *i* of respondent *j* condom use was reported as ‘never’, *Yij* = 2 for ‘rarely’, *Yij* = 3 for ‘sometimes’, *Yij* = 4 for ‘often’, and *Yij* = 5 for ‘always’.

### Statistical analysis

Pearson correlations were computed to get a basic insight about patterns of association of all variables. The distributions of the variables were assessed carefully for missing values and outliers, as these might unduly affect the results. Frequency of sex work had three values larger than 20 (two values 25, one 60) which were considered outliers; these were truncated to 20.

Taking into account the FSW as well as the sexual partners implies a multilevel structure [37], with the sexual partners, as network members, nested in FSWs as respondent level. The dependent variable, condom use, is a variable at the partner level. As explained below, the analysis proceeds in steps according to a conceptual framework summarizing the role of individual and network characteristics for HIV risk behaviors of FSWs. This framework is based on previous studies of the effects of various factors on HIV risk behaviors [14, 38–40] and goes from the more general background characteristics to the social network characteristics.

Responses about network members of the same respondent are likely to be correlated. Therefore, we used multilevel analysis [37, 41]. The number of respondents (called ‘level-2 units’ in the terminology of multilevel analysis) was 170; the total number of sexual network members (‘level-1 units’) was 615. We created a group mean (for groups defined as all sexual network members of a given FSW) for each explanatory variable at the level of the sexual partner, including age of sexual partner, education of sexual partner, frequency of contact, duration of tie, intimacy, social support, and drug or alcohol use before or with sex. This is required to investigate the difference between within-group and between-group regressions [37]. The within-group regression coefficient is the estimated parameter at the partner level, the between-group regression coefficient is the sum of the FSW-level and the partner-level coefficients.

Since the distribution of the dependent variable is highly skewed, with five values, we employed a multilevel ordered logistic regression model for ordered categorical outcomes [37]. This was the multilevel proportional odds model, which can be formulated as threshold model with *C* – 1 thresholds where *C* is the number of categories of the outcome variable; here *C* = 5. The mathematical expression of the model is

$${\displaystyle \begin{array}{c}\mathrm{P}\left\{{Y}_{ij}=c\right\}=\mathrm{P}\left\{{\theta}_{c-1}<{\tilde{Y}}_{ij}\le {\theta}_c\right\}\\ {}{\tilde{Y}}_{ij}={\sum}_h{\beta}_h{x}_{hij}+{U}_{0j}\end{array}}$$

Here *Y*_*ij*_ is the observed dependent variable for network member *j* of respondent *i*; *c* is an outcome ranging from 1 to 5; and P indicates probability. *Ỹ*_*ij*_ is a hypothetical unobserved auxiliary variable, which can be regarded as an underlying continuous variable that is observed after categorization according to thresholds *θ*_*1*_, *θ*_*2*_, *θ*_*3*_, and *θ*_*4*_. The observed outcome is *c* when *Ỹ*_*ij*_ is between the two thresholds *θ*_*c-1*_ and *θ*_*c*_, where the two outer thresholds formally are defined as minus or plus infinity: *θ*_*0*_ *= – ∞* and *θ*_*5*_ *= +∞*. The *β*_*h*_ are regression parameters; finally, the *x*_*hij*_ are the explanatory variables, which cover characteristics of respondents *i* as well as of sexual network members *j*; and *U*_*0j*_ is a respondent-level random effect with a standard logistic distribution.

We calculated the intraclass correlation coefficient (ICC), a descriptive statistic that measures the proportion of total variance of an outcome that is accounted for by the group level; in this case, the groups in the data refer to the FSWs. In other words, the ICC measures similarity in condom use between sexual partners of the same FSW. It was calculated according to formula (17.26, page 311) in [37], taking the within-group variance equal to *π*^2^/3=3.29 (the variance of the logistic distribution).

The model selection utilizes a conceptual framework based on previous studies of the effects of various factors on HIV risk behaviors [14, 38–40] and goes from more general background characteristics to the social network characteristics. This framework distinguishes three groups of independent variables. The first group is composed of the individual background characteristics of the FSW and her sexual partners, as indicated by age, education, and number of supported people. The second group consists of HIV knowledge and the behaviors directly associated with sex work: its frequency, whether it is accompanied by drug or alcohol use, and HIV testing. The third group is composed of social network characteristics and psychosocial mechanisms through which these may affect condom use, regarded as a behavior protecting against HIV risk: personal network density, and the tie characteristics such as duration of the tie, frequency of contact, and intimacy, social support, social norms, and drug or alcohol use before or with sexual relationship. These three groups may be interpreted as reflecting a hypothetical causal ordering, but we use this as a framework guiding the analysis and do not rely on assumptions of causality. In the multilevel ordered logistic regression analyses, a stepwise model selection procedure was employed, in which the groups of variables were entered sequentially. This allowed estimating the effect of social network characteristics on condom use while controlling for individual background characteristics of the FSWs and their sexual partners. It started with the empty model which contained only the dependent variable and the threshold parameters. Micro soft Excel was used for data management (data entry, quality control and cleaning of quantitative data). The data was analyzed by the ordinal package [42] in the R statistical system [43] which allows fitting a variety of mixed effects models for categorical outcomes. *p*-values less than 5% were regarded as statistically significant.

## Results

The mean age of the participants was 34.4 years (SD=7.6). Of the participants, 71 (42%) had a high school or diploma degree. Most participants reported living in their personal home (69, 40%). The mean score of HIV knowledge was 78.2 (SD=19.2). Among the participants, 11 (6%) reported that they had been HIV tested and were HIV positive. The mean age of initiating sex work and the frequency of sex work, after truncating outliers in the latter to 20, were 24.1 (SD=6.6), and 10.2 (SD=6.4), respectively. Table 1 shows the socio-demographic characteristics of the participants in detail.

**Table 1:** Sociodemographic characteristics of the participants (*N*=170)

Characteristics		N (%) or mean (SD)	Min-max
**Age**		34.4 (7.6)	17-58
**Marital status**	Single	33 (19%)	
	Married	137 (81%)	
**Educational level**	Illiterate	5 (3%)	
	Just reading and writing	2 (1%)	
	Primary education	18 (10%)	
	secondary education	68 (40%)	
	High school or Diploma	71 (42%)	
	University degree	6 (4%)	
**Number of supported people by the FSWs**	Nobody	104(61%)	
	One person or more	66 (39%)	
**Place of living**	Homeless	47 (28%)	
	Living in others’ home	54 (32%)	
	Personal home	69 (40%)	
**Frequency of sex work in the last month (truncated)**		10.2 (6.4)	1-60
**History of HIV testing**	Yes	139 (82%)	
	No	31 (18%)	
**HIV knowledge**		78.2 (19.2)	0-100

As Table 2 shows, the distribution of condom use, the dependent variable, was highly skewed. For most of the partners, FSWs reported that they never used condoms in sexual relationships with them in the last month (*N*=346, 56%). Only for nine sexual partners (1.5%) did the FSW always use condoms.

**Table 2:** Distribution of condom use in sexual relationships between the FSWS and their sexual partners in the last month (*N*=615 FSW-partner pairs)

Condom use categories	N (%)
**Never**	346 (56%)
**Rarely**	116 (19%)
**Sometimes**	75 (12%)
**Often**	69 (11%)
**Always**	9 (2%)

The participants named 615 people as their sexual network members. Table 3 gives descriptions of their characteristics. The age of the sexual partners was significantly correlated with the age of the FSWs (Mean=36.7, SD=7.5 vs Mean=34.4, SD=7.6, *r*=0.64, *p* < 0.01**)**. Many of the sexual partners like the FSWs had a high school or diploma degree 194 (32%). Many of the sexual partners were married (280, 46%). The mean sexual network size and density were 3.6 (SD=1), and 0.5 (SD=0.3), respectively. Among the sexual partners 175 (28%) had more than one role (e.g., familial tie or drug use partner) in the social network of the FSWs, which indicates multiplexity in the network. The FSWs reported that they did not know the HIV status of 61% of their sexual partners before the sexual relationship. The mean of the perceived safe sex norm was 34.7 (SD=10.4).

**Table 3:** Sexual network characteristics of the FSWs (*N*=615)

Characteristics		N (%) or Mean (SD)	Min-Max
**Sexual network size**		3.6 (SD=1.0)	1-8
**Sexual network density**		0.5 (SD=0.3)	0-1
**Multiplexity**	**Yes**	175 (28%)	
	**No**	440 (72%)	
**Duration of tie (Month)**		47.2 (SD=44.3)	1-408
**Frequency of contact in the last month**		14.1 (SD=11.9)	1-30
**Social support**		3.0 (SD=1.1)	0-5
**Intimacy**		4.1 (SD=0.9)	0-5
**Drug or alcohol use before or with sex**			
**Yes**		260 (42%)^a^	
**No**		353 (58%)	
**Perceived safe sex norm**		34.7 (SD=10.4)	17-34

^a^Drug or alcohol use before or with sex data was missing for two participants (*N*=613)

In the multilevel ordered logistic regression analyses, the intraclass correlation coefficient (ICC) was = 15.51/(15.51+3.29) = 0.82, signifying high similarity in condom use between the sexual partners of a given FSW.

Table 4 presents the results of the multilevel ordered logistic regression analyses according to the three models. The results of the first model, predicting condom use with individual background characteristics of the participants and their sexual partners, showed that FSWs’ age, partners’ age, and partners’ education were positively associated with condom use. Also, the mean education of the FSW’s partners was positively and significantly associated with condom use. A one-point increase in the average of partners’ education increases by 1.83 the conditional log odds of condom use. However, the mean age of the partners was not significantly associated (Model 1). Model 2, which included variables related to individual sexual practices and HIV test, adjusting for individual characteristics of FSW and sexual partners, showed that frequency of sex work in the last month was negatively associated with condom use. One unit increase in HIV knowledge of the FSWs significantly increases the conditional log odds of condom use by 0.062, which may seem small, but the standard deviation of HIV knowledge was 19.2, therefore, one standard deviation increase in HIV knowledge multiplies the odds of condom use by exp(19.2*0.062)=3.3. HIV testing did not have a significant association with condom use (Model 2). The last model included the partner-related and other social network variables, adjusting for the earlier included variables. One unit increase in the perceived safe sex norm score significantly increases by 0.113 the conditional log odds of condom use. The standard deviation of perceived safe sex norm was 10.4, so an increase by one standard deviation of the perceived safe sex norm score multiplies the odds of condom use by 3.2 (Model 3). In this model, individual background characteristics of the FSWs and their partners including age of the participants, age and education of their sexual partners, and HIV knowledge, after adjusting for other variables, still showed significant associations with condom use (Model 3). The R^2^ values [37] for Models 1 to 3 were 44, 54, and 69%, respectively.

**Table 4:** Results of multilevel ordered logistic regression models

	Model 1		Model 2		Model 3	
**Threshold parameters**	**Threshold**	**S.E.**	**Threshold**	**S.E.**	**Threshold**	**S.E.**
**Threshold 1-2**	6.98	3.50	8.45	3.61	4.39	4.78
**Threshold 2-3**	9.22	3.51	10.72	3.62	7.13	4.80
**Threshold 3-4**	11.33	3.54	12.85	3.65	9.82	4.84
**Threshold 4-5**	17.55	3.73	18.72	3.86	16.63	5.05
**Fixed effect**	**Coefficient**	**S.E.**	**Coefficient**	**S.E.**	**Coefficient**	**S.E.**
**Age**	0.237**	0.054	0.185 **	0.049	0.15**	0.043
**Educational level of FSWs**	0.37	0.31	0.52!	0.29	0.21	0.25
**No of supported people by FSWs**	1.01	0.60	0.94!	0.55	-0.15	0.50
**Age of sexual partners**	0.041*	0.020	0.032	0.021	0.067**	0.022
**Group mean of sexual partners’ age**	-0.124	0.075	-0.102	0.069	-0.088	0.062
**Educational level of sexual partners**	0.83**	0.14	0.87**	0.14	0.83**	0.15
**Group mean of sexual partners’ educational level**	1.83**	0.40	1.10**	0.37	0.56	0.36
**Frequency of sex work in the last month**			-0.376**	0.089	-0.089!	0.053
**HIV knowledge**			0.062**	0.018	0.052*	0.022
**HIV testing**			-0.63	0.85	0.47	0.89
**Network density**					-1.26	0.77
**Frequency of contact**					-0.270**	0.098
**Group mean of frequency of contact**					0.075*	0.037
**Duration of tie**					-0.064**	0.012
**Group mean of duration of tie**					-0.014!	0.008
**Intimacy**					-0.50*	0.24
**Group mean of intimacy**					-0.64	0.43
**Drug or alcohol use before or with sex**					0.26	0.86
**Group mean of drug or alcohol use before or with sex**					-1.67	1.02
**Perceived safe sex norm**					0.113**	0.029
**Social support**					-0.60*	0.28
**Level-two random effect**	**Variance**		**Variance**		**Variance**	
**Intercept variance**	9.89		6.64		4.69	

Significance codes for fixed effects: ** *p*-value < 0.01, * *p*-value < 0.05, ! *p*-value < 0.10

## Discussion

An interesting descriptive finding was the similarity between socio-demographic characteristics of the FSWs and their sexual partners (homophilous sexual partners in network terminology), consistent with Newcomb’s study among MSM in 2013 [44]. Such a similarity may create feelings of mutual perception, trust, and emotional closeness, which may lead to unsafe sex behaviors. Another important descriptive finding was the marital status of the FSWs and their sexual partners: most of them were married. This finding is consistent with other studies, and highlights the bridging role of FSWs and their sexual partners in transmission of HIV to the general population. Our finding showed that condom use on average was rather low. This finding is consistent with previous studies [45, 46] and suggests that not only infection risk, but also pregnancy risk is not a big stimulus to use condoms among Tehran FSWs. A national bio-behavioral survey among Iranian FSWs in 2010 reported that about 35% of the FSWs had a history of lifetime abortion. This abortion figure suggests that the effective protection against pregnancy risk is not very strong [46]. One explanation for this finding is related to the social stigma and negative attitudes from the community towards FSWs which has constructed an environment that is hardly conducive to accessibility of HIV prevention, condoms, and reproductive health services such as contraception [47, 48].

According to the final model of the multilevel analysis (Model 3, Table 4), the age and HIV knowledge of the FSW as well as the age and the educational level of her sexual partner had a positive effect on condom use. As regards the behavioral and attitudinal variables, intimacy with the sexual partner, as well as the duration of the tie, and the frequency of contacts, all had negative effects on condom use. For most variables at the partner level, the only variable with a significant effect for the group mean was frequency of contact with the sex partner, for which the within-group regression coefficient was estimated as -0.270 and the between-group regression coefficient as 0.075-0.270 = -0.195. This means that FSWs used condoms less with the clients with whom they had more frequent contact. Also the average condom use for FSWs who saw their clients more frequently was larger than for other FSWs, but this effect was less strong that might be expected based on the differences between clients for any given FSW. As regards other characteristics of the social network as a whole, perceived social support from the sexual partners, and perceived safe sex norms in the network of peers had a positive effect on condom use.

One explanation of the effect of age may be that older people have more knowledge and experience especially about consequences of unsafe sex and risk behaviors, and therefore may act more conservatively and more frequently use condoms in their sexual relationships. A related finding by Schick et al. [49] is that American adults over 50 years old who were in situations that posed an increased potential for risk (e.g., an unknown partner history or STI/HIV status) were more inclined to use condoms in their sexual relationships. However, some other studies, especially in the general population, showed contrary results as adolescents and younger adults used more condoms in their sexual relationships than older adults [50]. These contrasts may be due to differences in the studied populations, methodological aspects, and also social and cultural contexts. Iranian society is characterized by specific cultural and political settings, within which religion has an important role; educational programs for adolescents and young people, especially in public settings such as schools, are not usual and meet with various social and cultural sensitivities. Therefore, adolescent and young people may have insufficient information and knowledge about HIV risk behaviors, sexually transmitted diseases, and risk prevention methods, compared to adolescent and young people in other countries. Another reason which is specifically related to the context of our study, the FSWs in Tehran, is that younger FSWs, especially those who have relationships with older FSWs or pimps, may have lower HIV risk perception, because they have not been informed sufficiently about HIV risks and are more strongly controlled by the older FSWs or pimps in their networks [51].

FSWs with more HIV knowledge reported more frequent condom use. However, in spite of their rather high mean HIV knowledge, many FSWs reported to rarely or never use condoms in their sexual relationships. This suggests that having HIV knowledge in itself is not sufficient for safe sex practices among FSWs. The positive effect of knowledge corresponds to the positive effect of the educational level of FSWs’ sexual partners. Sexual partners with a higher educational level may have more knowledge about healthy behaviors and consequences of unsafe sex (health literacy), and may better understand the necessity of condom use. This finding is consistent with previous studies [11, 12, 52] and highlights the importance of educational programs for FSWs’ partners to prevent HIV risk behaviors.

FSWs with more frequent sex work in the last month were less likely to use condoms. However, the statistical significance of this association was a borderline. Frequent sexual relationships may go along with having multiple sexual partners, which would add to the risk of HIV infection since having frequent sexual relationships may provide greater opportunity for HIV transmission due to increase the likelihood of unsafe sex and exposure to HIV infected partners. This finding is consistent with previous studies [53, 54].

Our findings showed that frequency of condom use varied with the sexual partners. The FSWs used condoms significantly less with partners whom they had known for a long time; had more frequent contacts with; with whom they perceived more intimate relationships; and from whom they obtained more social support. These findings are consistent with previous studies [27, 55–58]. However, only one of these is a quantitative social network study among FSWs with partner-level information [57]. One explanation is that these mechanisms may create mutual trust and increase feelings of emotional closeness relationships between the FSW and her sexual partner [59]. After a long-term and repeated partnership, the FSW may have become familiar with her sexual partner and feel she knows him well. This may lead to accepting his requests for unsafe sex. This may further depend on several reasons such as the socio-economic status of the FSW and her dependence on her sexual partner [57], considering condom use as a threat to the trust in an intimate relationship [58, 60], power-gender inequality, inability of the FSW to negotiate with her partner about safe sex, and fear of the consequences of conflicts with him. Further research is needed to better understand the association between duration of tie, frequency of contact, and intimacy with condom use in this at-risk population.

Also, in line with previous studies [61, 62], our findings indicated that FSWs who perceive that their network members, especially their peer friends, have positive attitudes about safe sex and condom use, reported to use condoms more than those who did not have such a perception. This finding is consistent with Peterson and colleagues [18] who reported that men who have sex with men in the high-risk group, compared with those in the no-risk group, perceived lower positive reactions about condom use among their sexual network members. This confirms that networks are an important context for understanding social norms [63] and suggests that when developing interventions to change behavioral norms, the network context should be taken into account. When trying to promote condom use among FSWs, it may be beneficial to consider not only the attitudes and behaviors of the FSWs themselves, but also the attitudes and behaviors of their network members, especially those of their peer friends, who are colleagues and friends at the same time and may have dual roles as competitors and support providers. Network-based interventions may be useful to modify network relationships so as to create a social environment that can facilitate sexual behavior changes [64, 65].

### Strengths and limitations

Our study was conducted on a large sample of FSWs and obtained partner-level information about sensitive behavioral aspects of sexual behavior. We found that condom use on average was rather low. Using a multilevel approach to include variables at the personal, the relational, and the network level, we were able to obtain a high value of *R*^*2*^ to explain condom use. Variables that stood out in the explanation were associated with age, education, perceived norms in the network, and several variables related to intimacy with the partner. This information may be useful for effective evidence-based HIV prevention interventions. However, our findings should be considered also in the light of three main limitations. First, our study was cross-sectional. Therefore, we cannot draw causal inferences. Second, our findings might be subject to measurement error because our data was self-reported and collected from the focal individual in the personal network about their network members. Finally, our findings cannot be generalized to all FSWs in the country because we recruited the participants using a non-random snowball sampling from one city in Iran.

## Conclusion

The findings highlighted that considering only the individual characteristics of FSWs is not sufficient and we should also consider factors at the network level, especially the role of sexual partners, on condom use among FSWs. FSWs who had high-quality interactions with their sexual partners also exhibited more unsafe sex in their sexual relationships. The results suggest that to effectively promote condom use it is necessary to employ combined intervention programs that focus on the FSWs themselves as well as members of their social network.

## Data Availability

The datasets used and/or analyzed during the current study are available from the corresponding author on reasonable request.
